# COVID-19 Infection Among Incarcerated Individuals and Prison Staff in Lombardy, Italy, March 2020 to February 2021

**DOI:** 10.1001/jamanetworkopen.2022.4862

**Published:** 2022-03-30

**Authors:** Sara Mazzilli, Lara Tavoschi, Alessandro Soria, Marco Fornili, Giorgia Cocca, Teresa Sebastiani, Giuditta Scardina, Cristina Cairone, Guglielmo Arzilli, Giuseppe Lapadula, Luca Ceccarelli, Nicola Cocco, Raffaella Bartolotti, Stefano De Vecchi, Giacomo Placidi, Leonardo Rezzonico, Laura Baglietto, Ruggero Giuliani, Roberto Ranieri

**Affiliations:** 1 Scuola Normale Superiore, Pisa, Italy; 2 Department of Translational Research and New Technologies in Medicine and Surgery, University of Pisa, Pisa, Italy; 3 Clinic of Infectious Diseases, San Gerardo Hospital, ASST Monza, Monza, Italy; 4 Department of Clinical and Experimental Medicine, University of Pisa, Pisa, Italy; 5 Infectious Diseases Service, Penitentiary Health System, San Vittore Prison Health Unit, Azienda Socio-Sanitaria Territoriale Santi Paolo e Carlo, Milan, Italy; 6 School of Medicine and Surgery, University of Milano-Bicocca, Monza, Italy; 7 Welfare General Directorate, Lombardy Regional Health Authority, Milan, Italy

## Abstract

**Importance:**

Owing to infrastructural and population characteristics, the prison setting is at increased risk for transmission of SARS-CoV-2 and for severe clinical outcomes. Because of structural and operational reasons, research in prison settings is challenging and available studies are often monocentric and have limited temporal coverage; broader-based research is necessary.

**Objectives:**

To assess the extent and dynamics of the COVID-19 pandemic within the prison system of a large Italian region, Lombardy, and report the infection prevention and control measures implemented.

**Design, Setting, and Participants:**

This repeated cross-sectional study was carried out from March 1, 2020, through February 28, 2021 (first wave, March-June 2020; second wave, October 2020-February 2021) in the prison system of Lombardy, which includes 18 detention facilities for adults. All incarcerated persons and the prison staff of the penitentiary system of the Lombardy region participated in the study.

**Exposures:**

The main exposures of interest were the weekly average number of incarcerated individuals placed in quarantine in single or shared isolation rooms, the rate of sick leave by symptomatic and asymptomatic prison staff reported to the prison occupational medicine department on a weekly basis, and the level of overcrowding.

**Main Outcomes and Measures:**

The primary outcome measures were weekly COVID-19 crude case rates, weekly test positivity rate, and the relative risk of acquiring the infection for prison staff, incarcerated persons, and the general population.

**Results:**

The study population comprised a mean of 7599 incarcerated individuals and 4591 prison staff. Approximately 5.1% of the prison population were women; demographic characteristics of the prison staff were not available. During the study, COVID-19 occurred in 1564 incarcerated individuals and 661 prison staff. Most of these cases were reported during the second wave (1474 in incarcerated individuals, 529 in prison staff), when stringent measures previously enforced were relaxed. During both epidemic waves, incarcerated individuals and prison staff had a higher relative risk for COVID-19 infection than the general population during both the first wave (incarcerated individuals: 1.30; 95% CI, 1.06-1.58; prison staff: 3.23; 95% CI, 2.74-3.84) and the second wave (incarcerated individuals: 3.91; 95% CI, 3.73-4.09; prison staff: 2.61; 95% CI, 2.41-2.82).

**Conclusions and Relevance:**

The findings of this study suggest that the prison setting was an element of fragility during COVID-19 pandemic, with a high burden of COVID-19 cases among both the incarcerated individuals and prison staff. The prison setting and prison population need to be included and possibly prioritized in the response during epidemic events.

## Key Points

**Question:**

What was the COVID-19 infection rate among incarcerated individuals and staff during the first pandemic year in the prison system in Lombardy, Italy, when infection prevention measures had been implemented?

**Findings:**

In this cross-sectional study including a mean population of 7599 incarcerated individuals and 4591 staff members of 18 prisons in Lombardy, case rate and relative risk were significantly higher in incarcerated individuals and prison staff than the general population. Significant correlations between assessed control measures and case rate in incarcerated individuals were not found.

**Meaning:**

The findings of this study suggest that prison settings need to be included and prioritized in the framework of emergency preparedness and response.

## Teaser

This cross-sectional study examines the incidence of COVID-19 in the prison population vs the general population in Lombardy, Italy.

## Introduction

On February 20, 2020, the first major COVID-19 outbreak in Europe was detected in the Lombardy region, Italy.^1^ The Lombardy region is home to a sixth of the Italian population (10.03 million inhabitants^2^) and accounted for 20% of the COVID-19 cases and 27% of deaths in Italy, as of June 23, 2021.^3^ The temporal course of the epidemic was characterized by 3 distinct phases: the first epidemic wave was from March to June 2020, followed by a summer period with a relatively low incidence, and a second wave that started in September and peaked in November 2020.^3^

Evidence has shown that the risk of transmission of SARS-CoV-2 is much higher in prisons and other closed settings.^4^ Multiple large outbreaks of COVID-19 have been documented in detention facilities worldwide.^5,6^

Individuals in contact with the criminal justice system often come from marginalized groups of society with a higher burden of poverty and discrimination, and with limited access to health care, who represent the COVID-19 syndemic concept, for which the biological, economic, and social interactions between noncommunicable diseases and COVID-19 increase a person’s susceptibility to infection and worse health outcomes.^7^ The high turnover of people coming from the most disadvantaged segments of the population, together with the daily inflow and outflow of prison staff, increase the risk of virus introduction within prison. Once the virus enters the detention facilities, proximity, overcrowding, and infrastructural constraints facilitate the spreading of the infection.^8^

Italy has the highest prison density in the European Union, with an occupancy rate of 120%, and old penitentiary infrastructures.^9^ According to the most recent available data, incarcerated individuals in Italy are usually men who smoke (80%), with disproportionately higher rates of acute and chronic physical and mental illnesses compared with the general population, including diabetes, cardiovascular disease, and chronic respiratory diseases.^10^ Therefore, incarcerated individuals are more likely to experience severe COVID-19.

Lombardy had 7766 incarcerated persons as of February 28, 2021.^11^ During the first pandemic wave, Italian detention facilities implemented a number of preventive interventions, including (1) hygiene and sanitary measures (ie, use of personal protective equipment, handwashing, and environmental disinfection); (2) infection prevention and control measures (triage and COVID-19 test for new arrivals and isolation for suspected or confirmed cases); (3) measures to restrict access to essential staff, banning visitors, and discontinuing activities that required contact with individuals and communities from outside the prison; and (4) early release and alternatives to incarceration for low-risk offenders.^12,13^ With the conclusion of the first epidemic wave, the restrictive measures were gradually lifted, with the exception of hygiene and infection prevention and control measures,^14,15^ although with considerable variability between detention facilities.

In this study, we aimed to describe the progression of the COVID-19 epidemic in prison settings in Lombardy, Italy, during the first pandemic year (March 2020-February 2021), and report the infection prevention and control measures implemented during the same period.

## Methods

### Study Setting

In Lombardy there are 18 detention facilities for adults: 14 are correctional facilities where pretrial detainees and individuals with sentences shorter than 3 years are incarcerated and 4 are detention facilities for individuals with sentences longer than 3 years. The study was conducted at the request of regional authorities. This analysis used routine monitoring data collected in collaboration with the local health authorities for the purpose of containing the COVID-19 outbreak. This is standard operating procedure for medical interventions during public health emergencies. Authorization was obtained from the Ministry of Justice before data collection. Privacy and confidentiality of the patients were ensured. All data were anonymized or aggregated when entered into the database and the Ministry of Justice determined it was not necessary to obtain informed consent from individuals. No ethnic or other sensitive identifying information was encoded. This retrospective description of program data is exempt from review by the ethical review board. This study followed the Strengthening the Reporting of Observational Studies in Epidemiology (STROBE) reporting guideline for cross-sectional studies.

### Data Collection

#### COVID-19 Prison Data

COVID-19 data covering the period March 1, 2020, to February 28, 2021, were sourced from daily reports produced by each prison and submitted to the Prison Superintendence of the Lombardy region for surveillance purposes. The following variables were available: confirmed cases of COVID-19 among incarcerated persons, number of nasopharyngeal swabs, daily number of incarcerated persons in quarantine in single or shared cells, confirmed cases of COVID-19 among prison staff, and registered sick leaves taken by symptomatic and asymptomatic prison staff.

A detailed analysis of an intrafacility outbreak was performed for selected prisons (Milan Opera, a detention facility for long-sentenced individuals, and Milan San Vittore and Monza, correctional facilities for pretrial and short-sentenced individuals) during the first^12^ and second epidemic waves. We accessed routine clinical registries to source additional data including infection outcome, defined as the presence of symptoms compatible with COVID-19, hospitalization, and death.

#### Prison Population Data

Prison population was defined as the number of individuals detained in each imprisonment facility on the last day of the month as a proxy of the monthly number of individuals in prison. We used the mean of the number of individuals detained in each imprisonment facility on the last day of the month to calculate the average annual number of incarcerated persons. We acquired data on prison staff employed in each detention facility from the regional social security/occupational health database as of March 1, 2020, as a proxy for the entire study period.

#### COVID-19 General Population Data

The total number of inhabitants in Lombardy was sourced from the Italy National Institute of Statistics.^2^ COVID-19 data on the general population of Lombardy were obtained from a publicly accessible data set from the GitHub repositories developed by the Italian Presidency of the Council of Ministers and the Italian Department of Civil Protection.^16^ The following variables were available: confirmed cases of COVID-19 among incarcerated individuals and number of nasopharyngeal swabs performed.

### Statistical Analysis

For data analysis, we used Stata, version 13.0 (StataCorp LLC) and R, version 4.0.5 (R Foundation for Statistical Analysis). The number of confirmed cases minus the number of individuals who recovered or died was defined as active cases, which were considered the number of COVID-19 cases that were still infectious. We examined the weekly COVID-19 crude case rates per 1000 for prison staff, incarcerated individuals, and the general population. We examined the weekly testing rate (number of executed nasopharyngeal swabs in 1000 individuals) and the weekly test positivity rate (percentage of positive test results) in the incarcerated individuals and the general population. We calculated the weekly average number of incarcerated individuals placed in quarantine in single and shared isolation rooms per 1000 inmates. We determined the rate of sick leave used by symptomatic and asymptomatic prison staff reported to the prison occupational medicine department on a weekly basis. We assessed the level of overcrowding by calculating the percentage of individuals detained exceeding the regulatory capacity of the facility (9 m^2^ per person). We calculated the relative risk of acquiring the infection of incarcerated persons and prison staff compared with the general population during the first (March-June 2020) and the second (October 2020-February 2021) epidemic waves.

For the second epidemic wave (October 2020-February 2021), we studied the time series of the daily number of new COVID-19 cases among incarcerated individuals; daily number of new COVID-19 cases among staff members, incarcerated individuals in preventive isolation in single or shared rooms, and new asymptomatic or symptomatic sick leaves among staff. For each time series, data from all prisons were aggregated. To remove the long time trend from each of these series, negative binomial regression models were fitted with the effect of time modeled by B-spline functions (eFigure 1, eFigure 2 in the Supplement). The number of degrees of freedom of B splines was selected with the Akaike information criterion. The total number of incarcerated individuals or staff members were used as offsets. The deviance residuals of each negative binomial regression model were extracted, and the Spearman correlation coefficients were estimated for each couple of time series. The deviance residuals were fitted with linear models to estimate the lagged effect of each variable and the synchronous and lagged effects of the others.

The exposure variable overcrowding was available on a monthly basis and it presented little variability over time compared with the variability by prison; therefore, the outcome of overcrowding associated with the incidence of new cases among incarcerated individuals was evaluated using prison-specific data instead of prison-aggregated data. The monthly incidence of new cases among incarcerated individuals was modeled with a negative binomial model with the person-days of incarcerated individuals as offset and month as factor; the deviance residuals were then extracted and fitted with a linear model including overcrowding as a predictor. All statistical tests were 2-sided and *P* values less than .05 were considered as statistically significant.

## Results

From March 1, 2020, to February 28, 2021, an annual average of 7599 individuals (of whom approximately 5.1% were women) were detained in the 18 prisons of Lombardy, where 4591 prison staff were employed. Demographic characteristics of the prison staff were not available. The overcrowding rate of the imprisonment system of Lombardy was 131% at the start of the pandemic, decreased to 119% in April 2020, and gradually increased to 126% in October 2020, remaining stable until the end of the study observation period (eFigure 3 in the Supplement). The overcrowding rate was heterogeneous among the different prisons (range, 91%-202%), with all prisons exceeding the overcrowding rate of 100% for at least a month during the study period.

Between March 1, 2020, and February 28, 2021, a total of 1564 cases of COVID-19 were reported among incarcerated individuals in the Lombardy region, and additional 661 cases developed among prison staff. At the end of the first wave, 90 confirmed cases of COVID-19 were reported among incarcerated individuals and 132 cases among prison staff. Two of the 18 prisons reported no cases of COVID-19 among incarcerated individuals or prison staff. In 6 prisons, although cases of COVID-19 were reported among prison staff, no infections were detected among incarcerated individuals. From July 1, 2020, to February 28, 2021, 1474 confirmed cases of COVID-19 were reported among incarcerated individuals and 529 cases among prison staff. At least 1 case of COVID-19 was recorded among both prison staff and incarcerated individuals in all prisons (eFigure 4 in the Supplement).

### Epidemic Trends

**Figure 1.**
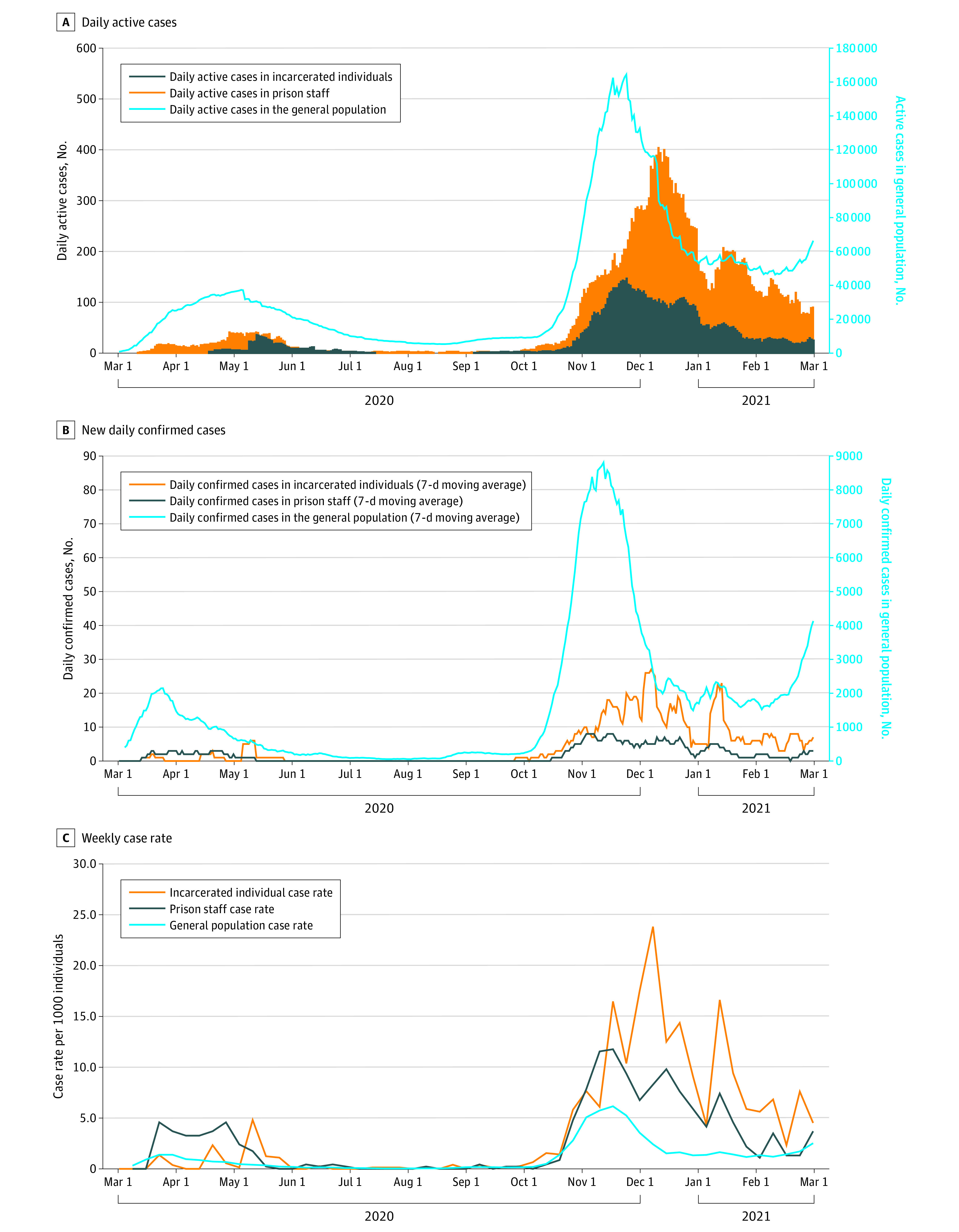
COVID-19 Cases Among Incarcerated Individuals, Prison Staff, and the General Population Trends from March 1, 2020, to February 28, 2021, in daily active cases (A), new daily confirmed cases (B), and weekly case rates (C).

During the first epidemic wave, there was a lag time of approximately 10 days between the start of the epidemic in the general population and the start of the epidemic in incarcerated individuals. The temporal gap between the epidemic peak in the general population and incarcerated individuals was more than 30 days (Figure 1A, B). Regarding prison staff, the trend of the epidemic curve of the first wave was unusual owing to suboptimal levels of access to diagnostic materials during the initial weeks of the outbreak, as described elsewhere.^12^

In incarcerated individuals and prison staff, the second epidemic wave began after a lag time of 15 days compared with the general population. The number of new cases per day in the general population and in prison staff reached its peak in the first 2 weeks of November 2020. The epidemic curve in incarcerated individuals was characterized by 2 peaks: the first occurred on December 5, 2020, and the second on January 9, 2021 (Figure 1B).

### Case Rates

Incarcerated individuals and prison staff had a higher relative risk for COVID-19 infection than the general population during both the first wave (incarcerated individuals: 1.30; 95% CI, 1.06-1.58; prison staff: 3.23; 95% CI, 2.74-3.84) and the second wave (incarcerated individuals: 3.91; 95% CI, 3.73-4.09; prison staff: 2.61; 95% CI, 2.41-2.82) (Figure 1C).

During the first wave, the relative risk of acquiring the infection compared with the general population was 1.30 (95% CI, 1.06-1.58) for incarcerated individuals and 3.23 (95% CI, 2.74-3.84) for prison staff. During the second wave, the relative risk of acquiring the infection compared with the general population was 3.91 (95% CI, 3.73-4.09) for incarcerated individuals and 2.61 (95% CI, 2.41-2.82) for prison staff.

According to the linear model using the deviance residuals, when the mutual synchronous and lagged effects were accounted for, the number of new cases among incarcerated individuals and new cases among staff presented a synchronous positive association (coefficient, 0.17; 95% CI, 0.02-0.33; *P* = .03). These results were confirmed by the Spearman correlation coefficient (Table).

**Table.** Spearman Correlation Coefficients Between the Residuals of Each Time Series Under Different Negative Binomial Regression Models^a^

Characteristic	New cases among incarcerated individuals	New cases among staff	Quarantined incarcerated individuals
New cases among staff			
Model 1	0.48^b^	NA	NA
Model 2	0.24^b^	NA	NA
Incarcerated individuals in quarantine			
Model 1	0.41^b^	0.43^b^	NA
Model 2	–0.15	–0.08	NA
New sick leave among staff			
Model 1	0.24^b^	0.36^b^	0.28^b^
Model 2	–0.01	0.02	0.22^b^

Abbreviation: NA, not applicable.

^a^ Model 1, time series correlation; model 2, time series correlation after removing the long-term time association.

^b^ *P* < .01.

### Testing and Positivity Rates

During the first epidemic wave, the mean weekly testing rate per 1000 individuals was 61.09 (range, 0-115.44) in incarcerated individuals and 6.11 (range, 1.16-10.41) in the general population. During the second wave, the average weekly positivity rate was 258.43 (range, 123.92-573.08) in incarcerated individuals and 19.73 (range, 11.68-30.09) in the general population (eFigure 5 in the Supplement). During the first epidemic wave, the average weekly positivity rate per 100 individuals was 1.76 (range, 0.00-10.68) in incarcerated individuals and 9.55 (range, 1.21-37.50) in the general population. During the second wave, the average weekly positivity rate was 4.46 (range, 0.00-17.92) in incarcerated individuals and 8.71 (range, 1.16-20.71) in the general population (eFigure 6 in the Supplement).

### Containment Measures

**Figure 2.**
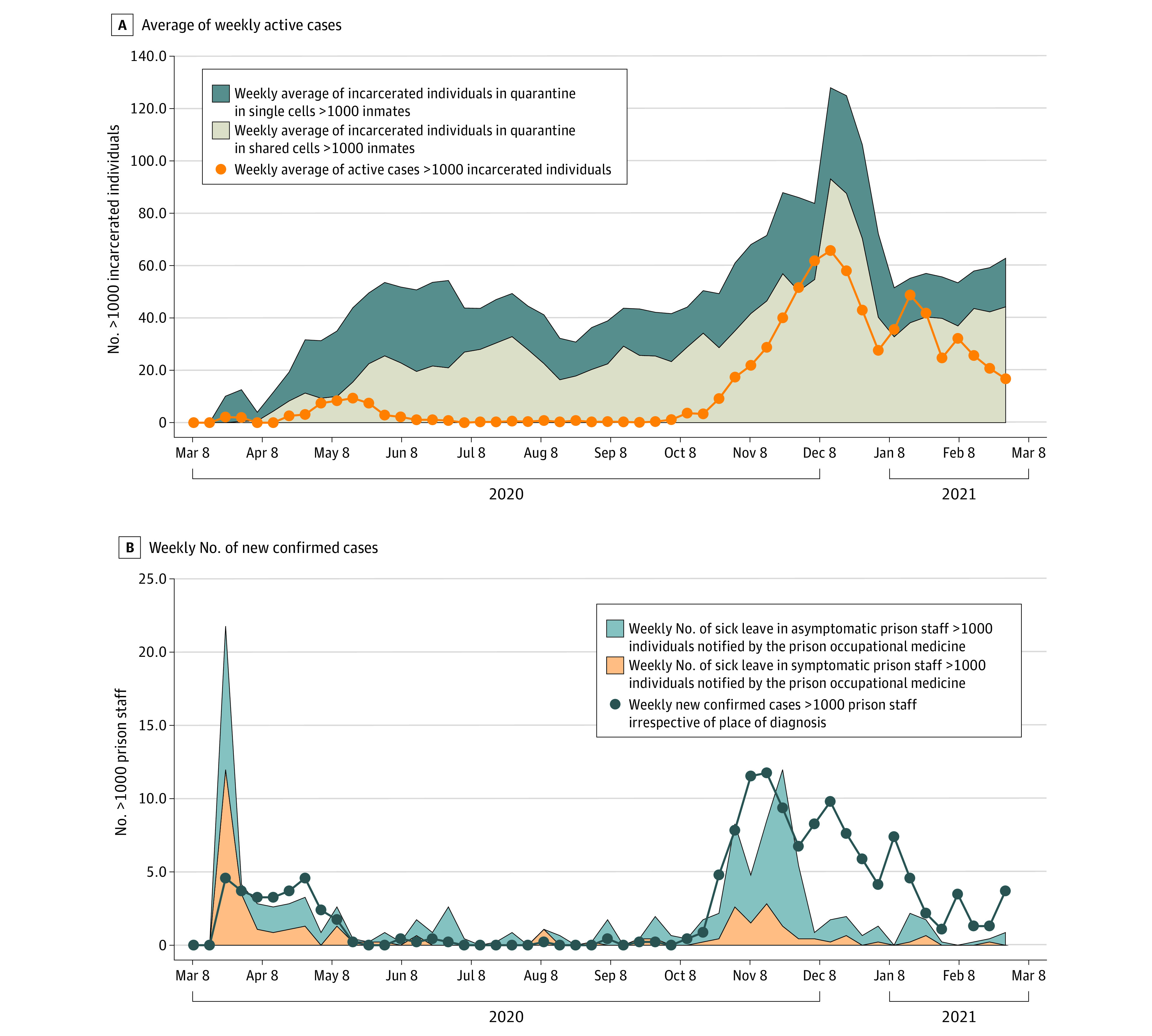
Containment Measures Trends from March 1, 2020, to February 28, 2021, in incarcerated individuals in shared cells vs solitary confinement (A) and new confirmed cases by sick leave among asymptomatic and symptomatic prison staff (B).

The measure of quarantine in single or shared cells was introduced from April 5, 2020, for incarcerated individuals who had suspected or confirmed COVID-19. The weekly average number of incarcerated individuals placed in quarantine increased until the end of June and then had an irregular trend until the start of the second wave. Quarantine in single or shared cells was used evenly over time, except for the second epidemic wave, when an increase in active cases (Figure 1A) led to an increase in incarcerated individuals quarantined in shared cells (Figure 2A).

At the beginning of the pandemic, the number of individuals who were asymptomatic and symptomatic on sick leave reported to the prison occupational medicine department was far greater than the number of confirmed cases (Figure 2B). Starting in April 2020, the number of staff on sick leave decreased and became comparable with the number of confirmed cases. However, during the second wave, the number of asymptomatic and symptomatic individuals on prison occupational medicine registered sick leave was lower than the number of confirmed cases (Figure 2B).

According to the linear model using the deviance residuals, no statistically significant association was observed between the incidence of new cases among incarcerated individuals and prison-specific overcrowding (coefficient, 0.0030; 95% CI, −0.0044 to 0.0103; *P* = .43). The Spearman correlation coefficient among containment measures (the daily number of incarcerated individuals in preventive isolation in single or shared rooms; the daily number of new asymptomatic or symptomatic sick leaves among staff) and the daily number of new COVID-19 cases among incarcerated individuals and staff members was significant when the long-term time outcome was not removed, but became nonsignificant after removing the long-term time effect (Table).

### Insights From 3 Prison Outbreaks

**Figure 3.**
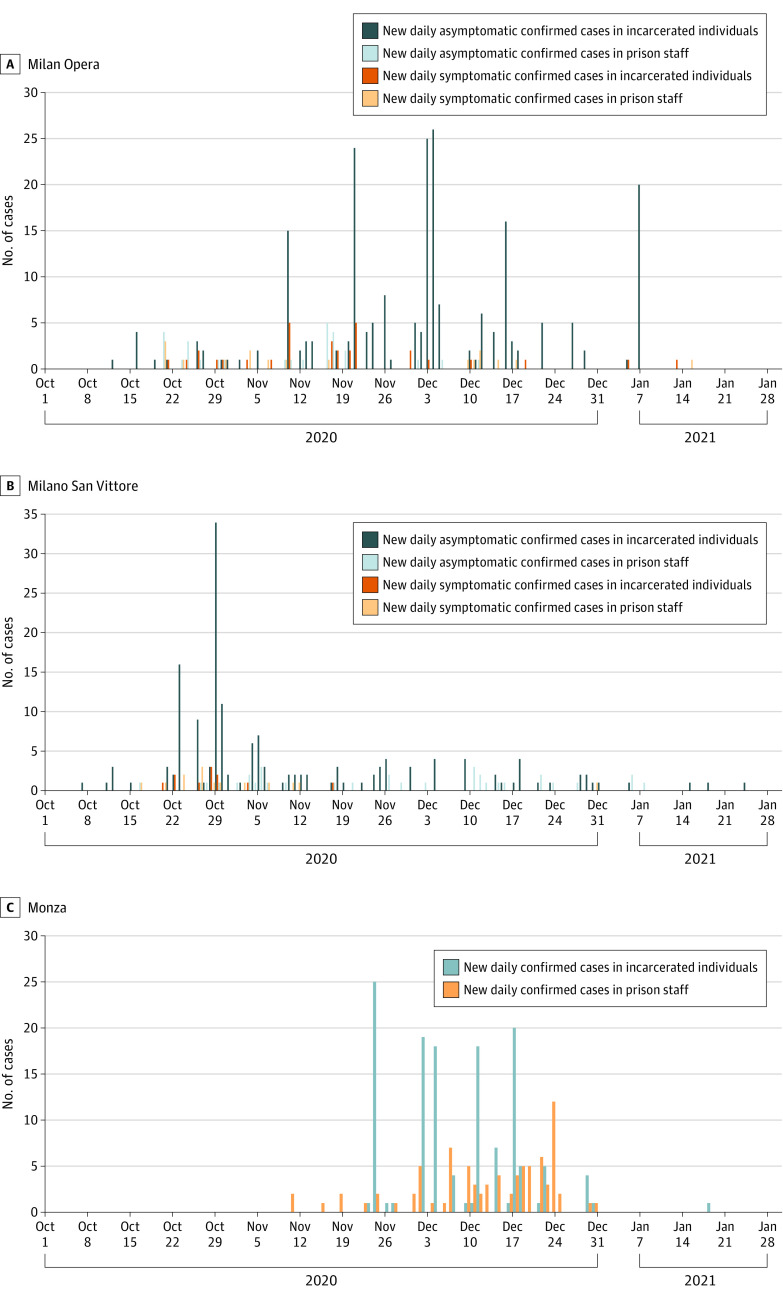
Daily Confirmed Cases in Incarcerated Individuals and Prison Staff in 3 Prisons in the Lombardy Region Confirmed cases of COVID-19 in the Milan Opera (A), Milan San Vittore (B), and Monza (C) prisons.

Between October 2020 and January 2021, in Milan Opera, 253 cases of COVID-19 were confirmed among incarcerated individuals; 12 persons were hospitalized and 1 died, and 45 cases of COVID-19 were reported among prison staff. In Milan San Vittore, 165 cases were confirmed among incarcerated individuals; 10 people were hospitalized and 52 cases were detected among prison staff. In Monza, 134 cases were confirmed among incarcerated individuals; 2 people were hospitalized and 1 died (for reasons other than COVID-19), and 83 cases were detected among prison staff (Figure 3). The occurrence and progression of the 3 outbreaks were heterogeneous and prison specific. The proportion of symptomatic confirmed cases was 12.65% in incarcerated individuals and 37.78% in prison staff in Milan Opera prison; the proportions in San Vittore prison were 6.67% in incarcerated individuals and 34.62% in prison staff.

## Discussion

Herein we describe the extent and dynamics of COVID-19 spread within the penitentiary system of Lombardy, the most populated Italian region, during the first 12-month of the pandemic. To our knowledge, this is the first attempt to extensively study COVID-19 in the prison setting in a European country. Although evidence from other countries is available, such as the US,^17^ important differences in health care, prison systems, and sociodemographic composition of the populations make it challenging to translate such findings into the European context.

Among Italian regions, Lombardy was one of the most affected by COVID-19 during both the first and second waves. Yet, the COVID-19 epidemic among incarcerated individuals was heterogeneous across the region and over time. During the first epidemic wave, COVID-19 spread within the prison system was largely contained and a limited number of cases occurred among incarcerated individuals.^12^ When comparing crude case rates, incarcerated individuals were in fact protected from the infection compared with residents in the region, even when considering the substantial level of underascertainment and underestimation that characterized the initial months of the pandemic.^18^ Incarcerated individuals had higher coverage of testing and screening compared with the general population during the first epidemic wave, as suggested also by the test positivity rates during that period. However, during the second epidemic wave, the burden of COVID-19 among incarcerated individuals was substantially higher, with greater than 10 times more cases and an overall crude case rate greater than that observed among the general population. The distribution of COVID-19 in incarcerated individuals over time was also distinctive, with multiple peaks, likely due to outbreak episodes within different prison institutions. These observations suggest that incarcerated individuals are at increased risk of infection from outbreak-prone airborne diseases.^19^ Indeed, detention facilities are often characterized by overcrowding, poor ventilation, poor access to health care services, and a high turnover of staff and incarcerated individuals, which are features that facilitate the entry and spread of the virus.^20^ Owing to the prevalence of underlying health conditions in the Italian context, incarcerated individuals are also at higher risk of developing severe clinical events when infected by SARS-CoV-2.^10,21^

The difference in the burden of COVID-19 among incarcerated individuals between the 2 waves may be attributed to multiple factors, including the different degree to which control interventions were implemented. Stringent measures were enforced early with the establishment of national lockdown, the banning of virtually all activities in prison settings, including visits, and prison de-population.^8,13^ Although effectively shielding incarcerated individuals from SARS-CoV-2 entering prisons, these measures triggered revolts and may have worsened the mental health of prison residents.^8,13,22^ Since June 2020, containment measures were progressively relaxed in community and prison settings, with various degrees of limitation to interprison and prison/community movement being maintained throughout the period. Also, the rates of incarceration, occupancy, and overcrowding returned to pre–COVID-19 levels during the second epidemic wave, possibly increasing the overall risk of virus introduction and spreading within prison facilities.^23^

We did not find any significant correlation between assessed control measures and the daily number of new COVID-19 cases among incarcerated individuals during the study period. However, a previous assessment focused on the first wave showed an association between the number of cases and quarantine in single rooms.^12^ During the second wave, the quarantine was mainly implemented in shared rooms owing to space constraints—a practice that may have affected COVID-19 incidence. Infrastructural constraints, old buildings, and overcrowding hinder successful management of COVID-19 and potentially the outbreak of other communicable diseases in prison settings. Also, similar bundles of measures may have been implemented with different modalities or intensity in different prison institutions for several reasons, including prison setup, availability of staff or commodities, and incarcerated individual characteristics (eg, long-term vs short-term sentence). These, and possibly additional contextual factors, such as level of virus circulation in the community or staff turnover, may have led to different distribution and intensity of intrafacility outbreaks.

Prison staff navigating between correctional facilities and their communities may experience multiple exposures to SARS-CoV-2. According to our results and other data,^24^ prison staff have an increased risk of testing positive for SARS-CoV-2 compared with the general population. From the distribution of cases over time, we observed that, during both the first and second epidemic waves, the spread of the virus in prison staff occurred earlier than in incarcerated individuals. Therefore, it is likely that prison staff played a role in introducing and spreading SARS-CoV-2 in detention facilities. The prison staff also may have multiple occasions for acquiring SARS-CoV-2 infection during their occupational duties, such as escorting detainees to health services or supervising hospitalized prisoners. Moreover, prison staff often live in the prison compound, sharing living space with colleagues.^25^ Intrafacility outbreak analysis showed a higher proportion of confirmed symptomatic cases in prison staff than in incarcerated individuals. Because incarcerated individuals have worse health status,^26^ this result was unexpected and may be explained by 2 factors. First, there may have been an underestimation of symptomatic cases in incarcerated individuals owing to the lack of a systematic and continuous recording of follow-up clinical data. Second, coverage of COVID-19 testing was lower among prison staff compared with incarcerated individuals. This disproportion was documented during the first wave.^12^ Asymptomatic cases among prison staff may have not been detected, further supporting the hypothesis that prison staff played a role in spreading SARS-CoV-2 into prison settings.

This study did not include any evaluation of the effect of COVID-19 and preventive measures on the mental health of detained persons. Further research in this area may provide a deeper understanding of the influence of the pandemic on the well-being of incarcerated individuals and an opportunity to better plan and manage future epidemic events occurring in prison settings. This research is particularly relevant because the availability of data on the direct effect of the COVID-19 pandemic on incarcerated individuals and prison staff was limited and not systematically collected or assessed.

### Limitations

This study has limitations. First, it was not possible to obtain information on individuals admitted, moved, or released from prison during the study period. Therefore, the number of individuals who were in contact with the Lombardy detention system was approximated and most likely underestimated. Second, the number of nasopharyngeal swabs performed in prison staff was not available and it was not possible to calculate the positivity rate. Third, during the second wave, prison staff also had access to the nasopharyngeal swabs outside the prison facility; therefore, confirmed cases in this population may have been underreported. Fourth, because the general population had lower test coverage compared with incarcerated individuals and, consequently, a potential underestimation of the COVID-19 burden, the relative risk of incarcerated individuals compared with the general population may be overestimated.

## Conclusions

Despite the fact that health care provisions in Italian prisons is in the remit of Ministry of Health,^27^ the lack of integrated health care and preventive services as well as surveillance activities between prison and community were elements of fragility in the pandemic response. The containment measures implemented during the first epidemic wave contributed to limit the spread of the infection, but these measures might have come with high social and emotional costs. The burden of COVID-19 among incarcerated individuals and prison staff during the second epidemic wave noted the challenges in implementing effective prevention and control measures in the prison settings, as well as the increased risks and vulnerability of such population groups to respiratory infections. Therefore, the prison settings and prison populations should be considered in response efforts during epidemic events.

## Notes

Supplement. **eFigure 1.** Trend of Number of New Daily Confirmed Cases Among Incarcerated Individuals From October 1 to February 28 **eFigure 2.** Trend of Number of New Daily Confirmed Cases Among Prison Staff From October 1 to February 28 **eFigure 3.** New Daily Confirmed Cases Among Incarcerated Individuals in the 18 Prisons of the Lombardy Region (March 1, 2020-February 28, 2021) **eFigure 4.** Overcrowding Level in the Penitentiary System of the Lombardy Region **eFigure 5.** Weekly Testing Rate Among 1000 Incarcerated Individuals and Among 1000 Individuals of the General Population **eFigure 6.** Weekly Positivity Rate Among Incarcerated Individuals and Among the General Population
