# Supplementary material for: COVID-19 Infection Among Incarcerated Individuals and Prison Staff in Lombardy, Italy, March 2020 to February 2021
Source: JAMA Netw Open. 2022 Mar 30;5(3):e224862. doi: 10.1001/jamanetworkopen.2022.4862 (PMC8968466; doi:10.1001/jamanetworkopen.2022.4862)
Supplement: Supplement. — eFigure 1. Trend of Number of New Daily Confirmed Cases Among Incarcerated Individuals From October 1 to February 28 eFigure 2. Trend of Number of New Daily Confirmed Cases Among Prison Staff From October 1 to February 28 eFigure 3. New Daily Confirmed Cases Among Incarcerated Individuals in the 18 Prisons of the Lombardy Region (March 1, 2020-February 28, 2021) eFigure 4. Overcrowding Level in the Penitentiary System of the Lombardy Region eFigure 5. Weekly Testing Rate Among 1000 Incarcerated Individuals and Among 1000 Individuals of the General Population eFigure 6. Weekly Positivity Rate Among Incarcerated Individuals and Among the General Population [file jamanetwopen-e224862-s001.pdf]

## Supplementary Online Content

Mazzilli S, Tavoschi L, Soria A, et al. COVID-19 infection among incarcerated individuals and prison staff in Lombardy, Italy, March 2020 to February 2021. *JAMA Netw Open*. 2022;5(3):e224862. doi:10.1001/jamanetworkopen.2022.4862

**eFigure 1.** Trend of Number of New Daily Confirmed Cases Among Incarcerated Individuals From October 1 to February 28

**eFigure 2.** Trend of Number of New Daily Confirmed Cases Among Prison Staff From October 1 to February 28

**eFigure 3.** New Daily Confirmed Cases Among Incarcerated Individuals in the 18 Prisons of the Lombardy Region (March 1, 2020-February 28, 2021)

**eFigure 4.** Overcrowding Level in the Penitentiary System of the Lombardy Region

**eFigure 5.** Weekly Testing Rate Among 1000 Incarcerated Individuals and Among 1000 Individuals of the General Population

**eFigure 6.** Weekly Positivity Rate Among Incarcerated Individuals and Among the General Population

This supplementary material has been provided by the authors to give readers additional information about their work.

© 2022 Mazzilli S et al. *JAMA Network Open.*

**eFigure 1**. Trend of number of new daily confirmed cases among incarcerated individuals from October 1 to February 28: observed values (black line), predicted values (red line), 95% confidence band (shaded pink area) and 95% predicted band (shaded grey area).

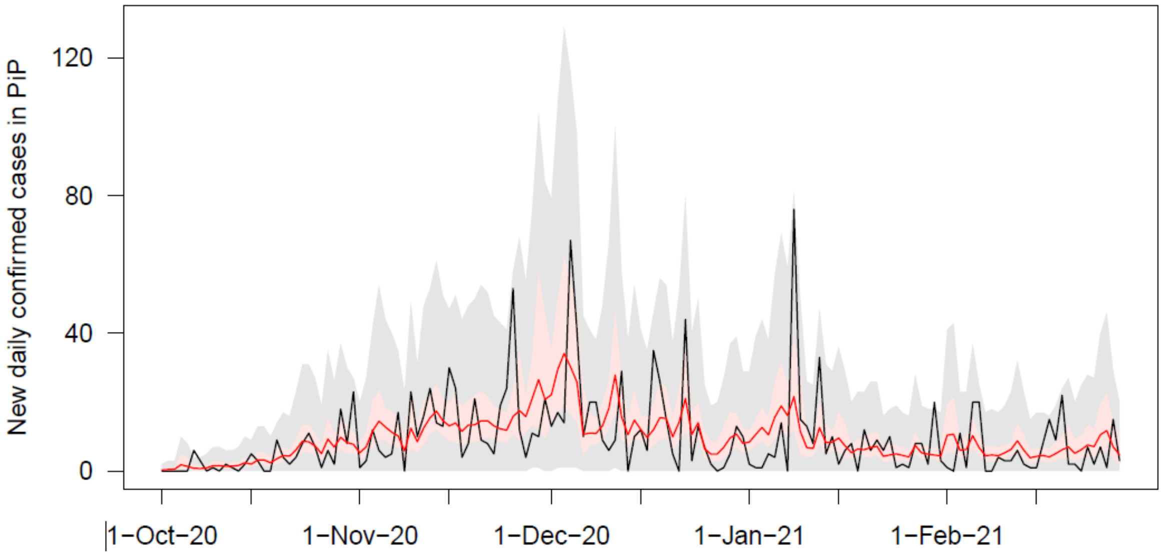


© 2022 Mazzilli S et al. *JAMA Network Open.*

**eFigure 2**. Trend of number of new daily confirmed cases among prison staff from October 1 to February 28: observed values (black line), predicted values (red line), 95% confidence band (shaded pink area) and 95% predicted band (shaded grey area).

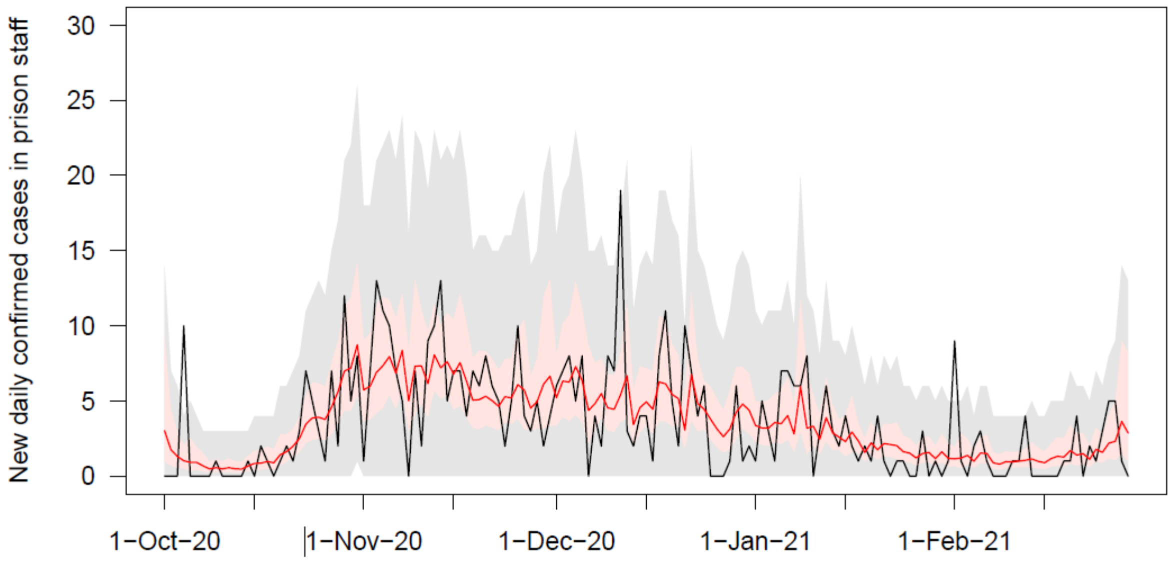


© 2022 Mazzilli S et al. *JAMA Network Open.*

**eFigure 3.** New daily confirmed cases among incarcerated individuals in the 18 prisons of the Lombardy region (March 1, 2020 – February 28, 2021)

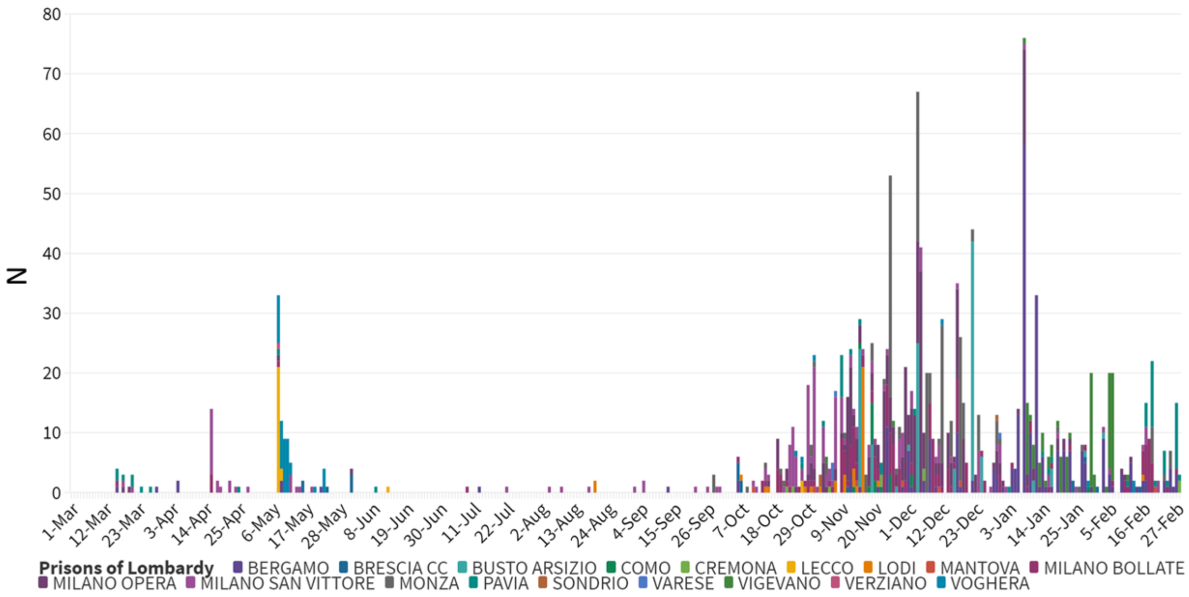


© 2022 Mazzilli S et al. *JAMA Network Open.*

**eFigure 4.** Overcrowding level in the penitentiary system of the Lombardy region.

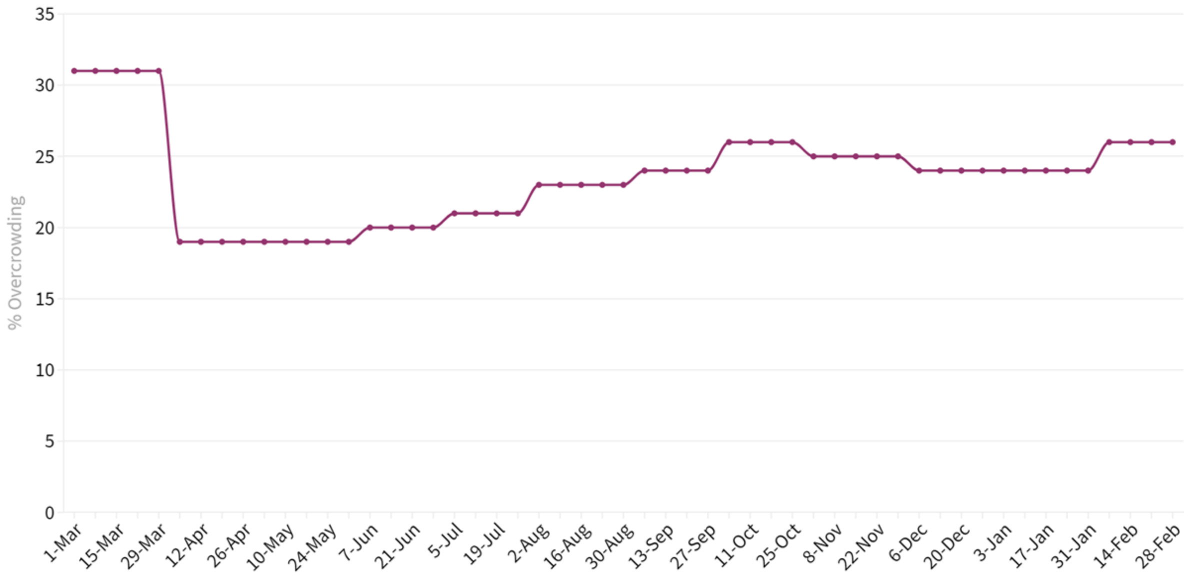


© 2022 Mazzilli S et al. *JAMA Network Open.*

**eFigure 5.** Weekly testing rate among 1000 incarcerated individuals and among 1000 individuals of the general population.

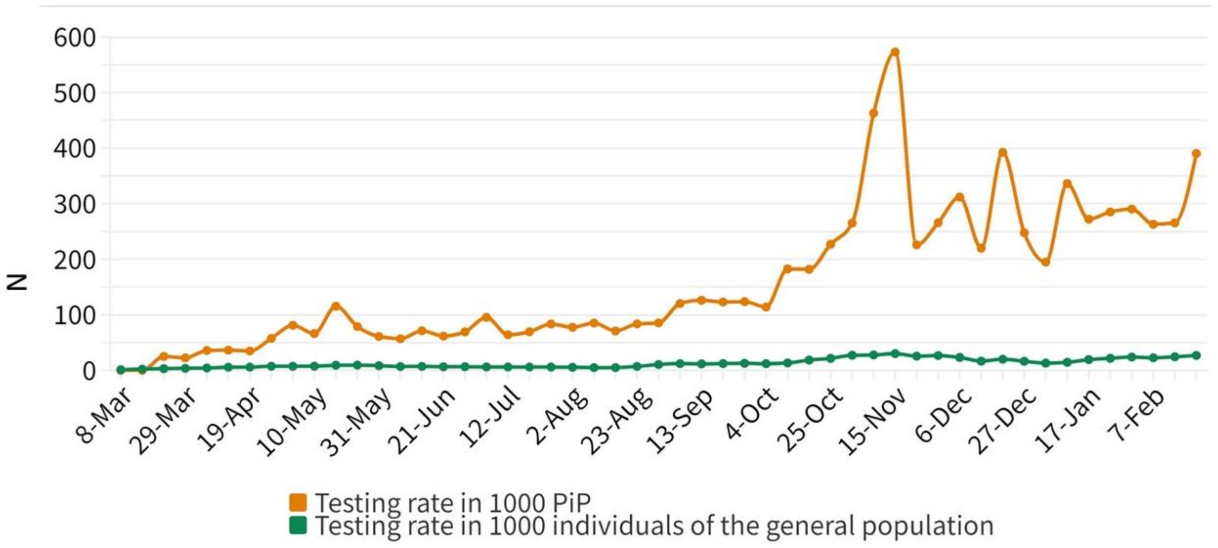


© 2022 Mazzilli S et al. *JAMA Network Open.*

**eFigure 6.** Weekly positivity rate among incarcerated individuals and among the general population.

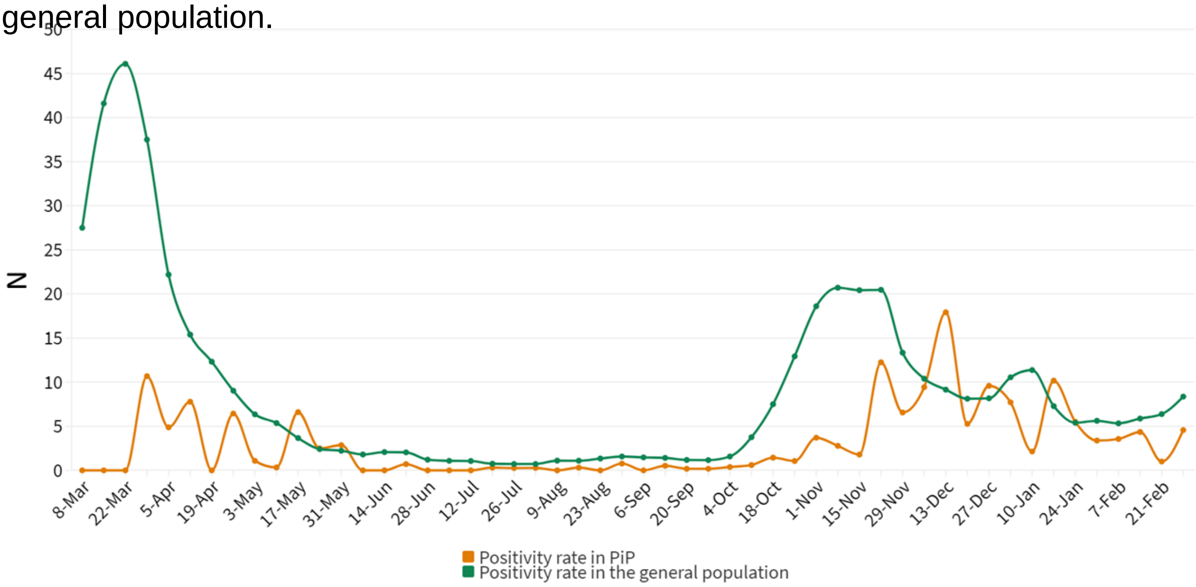


© 2022 Mazzilli S et al. *JAMA Network Open.*
